# Multilayer Double Emulsion Encapsulation of *Limosilactobacillus reuteri* Using Pectin-Protein Systems

**DOI:** 10.3390/foods14142455

**Published:** 2025-07-12

**Authors:** Kattya Rodríguez, Diego Catalán, Tatiana Beldarraín-Iznaga, Juan Esteban Reyes-Parra, Keyla Tortoló Cabañas, Marbelis Valdés Veliz, Ricardo Villalobos-Carvajal

**Affiliations:** 1 Department of Food Engineering, Faculty of Health and Food Science, Universidad del Bío-Bio, Av. Andrés Bello 720, P.0. Box 447, Chillán 3800708, Chile; kattyarodriguez49@gmail.com (K.R.); diego.catalan1801@alumnos.ubiobio.cl (D.C.); jreyes@ubiobio.cl (J.E.R.-P.); keyla.tortolo2301@alumnos.ubiobio.cl (K.T.C.); marbe.veliz@gmail.com (M.V.V.); 2 Department of Animal Science, Faculty of Veterinary Sciences, Universidad de Concepción, Av. Vicente Méndez 595, Chillán 3780000, Chile; tbeldarrain@udec.cl

**Keywords:** double emulsion, layer-by-layer electrostatic deposition, ionic gelation, viability, thermal treatment, simulated in vitro digestion, *Limosilactobacillus reuteri*

## Abstract

The development of bakeable foods supplemented with probiotics requires novel strategies to preserve the functionality of probiotic cells during thermal and gastrointestinal stress conditions. The objective of the present study was to evaluate the protective effect of multilayer double emulsions (W_1_/O/W_2_) stabilized with pectin-protein complexes on the viability of *Limosilactobacillus reuteri* (Lr) under thermal treatment (95 °C, 30 min), storage (4 °C, 28 d), and simulated gastrointestinal conditions. Emulsions were prepared with whey protein isolate (WPI) or sodium caseinate (Cas) as outer aqueous phase emulsifiers, followed by pectin coating and ionic gelation with calcium. All emulsions were stable and exhibited high encapsulation efficiency (>92%) with initial viable counts of 9 log CFU/mL. Double emulsions coated with ionically gelled pectin showed the highest protection against heat stress and gastrointestinal conditions due to the formation of a denser layer with lower permeability, regardless of the type of protein used as an emulsifier. At the end of storage, Lr viability exceeded 7 log CFU/mL in cross-linked pectin-coated microcapsules. These microcapsules maintained >6 log CFU/mL after thermal treatment, while viability remained >6.5 log CFU/mL during digestion and >5.0 log CFU/mL after consecutive heat treatment and simulated digestion. According to these results, the combination of double emulsion, multilayer formation and ionic crosslinking emerges as a promising microencapsulation technique. This approach offers enhanced protection for probiotics against extreme thermal and digestive conditions compared to previous studies that only use double emulsions. These findings support the potential application of this encapsulation method for the formulation of functional bakeable products.

## 1. Introduction

The composition of the gut microbiota plays a critical role in human health. Dysbiosis has been linked to several conditions, including obesity, diabetes, immune system disorders, asthma, and allergic rhinitis. Incorporating probiotics into the diet has emerged as a current strategy to restore microbial balance [1]. Probiotics are defined as live microorganisms that confer health benefits to the host when administered in adequate amounts [2]. Probiotic concentrations at the time of consumption should be at least 9 log CFU per serving to provide beneficial effects, ensure survival through gastrointestinal transit, and release a minimum of 6–7 log CFU/g in the intestine [3].

*Limosilactobacillus reuteri* (Lr) is a safe and effective bacterium to prevent and treat numerous gastrointestinal disorders, such as dysbiosis, diarrhea, constipation, or inflammatory bowel diseases [4,5]. However, probiotics are sensitive to environmental stressors, such as heat, oxygen, acidity, and bile salts [6]. Its direct incorporation into food can compromise their viability during processing, storage, and digestion [7,8]. Therefore, protecting probiotic cells to preserve their functionality remains a key challenge in the development of functional foods. Various strategies have been explored to enhance the stability of probiotic cells under stress conditions, with the microencapsulation process emerging as a practical method for protection [9]. Microencapsulation is a promising strategy for protecting probiotic bacteria under stress conditions. Probiotics are trapped within a semi- or non-permeable matrix or membrane material in microencapsulation [10].

Among the available encapsulation techniques, double emulsions (W1/O/W2) have shown promising results in protecting probiotics [6,11]. In these systems, probiotics are trapped in the internal aqueous phase (W_1_), surrounded by an oil phase (O), and stabilized by an external aqueous phase (W_2_). This structure provides a physical barrier that reduces exposure to harsh environments such as heat and gastrointestinal fluids [12,13]. Nevertheless, the thermodynamic instability of double emulsions is a limitation because coalescence and rupture can occur during storage or when exposed to stress conditions [14,15].

To address this challenge, strategies such as osmotic balance, interfacial thickening, and the use of biopolymeric emulsifiers have been explored to enhance emulsion stability [16,17]. Milk proteins are excellent emulsifiers because of their amphiphilic nature and ability to form interfacial films [18]. Whey protein isolate (WPI) and sodium caseinate (Cas) are food-grade proteins with distinct structural characteristics; WPI is a globular protein with high interfacial activity, while Cas can form flexible, multilayered films that depend on environmental pH [19,20]. These differences influence the physicochemical properties of emulsions and their protective efficacy [12,21].

Cas and WPI have been used in the outer aqueous phase of double emulsions to protect probiotics and improve their stability when exposed to gastrointestinal conditions [12,22]. In turn, due to their amphiphilic nature, these proteins can also be used in conjunction with anionic polysaccharides such as pectin to form multiple layers at the interface of the double emulsion. By controlling the pH of the medium, their electrical charges can be modified to obtain the maximum electrostatic charge difference between proteins and polysaccharides [23].

The formation of multilayers on encapsulated systems using the layer-by-layer electrostatic deposition technique with oppositely charged biopolymers is a strategy that provides enhanced protection for bioactive compounds against thermal treatment, acidic pH, and gastrointestinal conditions [24,25]. Saffron encapsulation using a double emulsion coated with a bilayer of whey protein and pectin increased the stability of saffron compounds under environmental stress conditions [26,27]. Although multilayer coatings have been widely studied in oil-in-water emulsions, their application in W_1_/O/W_2_ systems for probiotic delivery is still limited [17].

Low-methoxyl pectin (DE <50%) forms electrostatic complexes with proteins and gels when calcium ions are present, contributing to the mechanical strength and colon-targeted release of encapsulated probiotics [28,29]. The results of our previous study indicate that the ionic cross-linking of an external alginate layer formed on a double emulsion containing *Limosilactobacillus casei* could provide greater thermal resistance to the probiotics, due to an increase in the glass transition temperature and melting temperature of the microcapsules. This fact could allow the use of these microcapsules in the development of baking functional products and pasteurized juices [12].

Considering this background, this research will study the inclusion of probiotic cells in the internal aqueous phase of a double emulsion, since the limited migration of cells to the outside of the capsules has been proven, generating a high efficiency of encapsulation and reducing exposure to adverse environmental conditions [30]. In turn, an alginate layer will form on the external aqueous phase of the double emulsion, and this will undergo an ionic crosslinking process to create an additional physical barrier against gastric fluids, thereby enhancing the thermal stability of the probiotics [23].

Therefore, the objective of the present study was to evaluate the performance of Cas-pectin and WPI-pectin multilayer double emulsions to encapsulate *Limosilactobacillus reuteri*, a probiotic known for its beneficial effects on gastrointestinal health [4,5]. The study focused on assessing the encapsulation efficiency and stability of the emulsions, as well as the impact of the different interfacial structures formed on the protection of the probiotic against thermal and gastrointestinal stress, and storage conditions. It also provided insights into their potential application in functional food formulations.

## 2. Materials and Methods

### 2.1. Materials

The oil phase of the emulsions consisted of canola oil (O), which was purchased at a local supermarket and used without further purification. The WPI (protein 97%) was purchased from Winkler (Santiago, Chile). The Cas from bovine milk (C8654), Tween 80 (W291706), and Span 80 (85548) to form the multilayer double emulsions were obtained from Sigma Aldrich Co. (St. Louis, MI, USA). Calcium chloride (≥97%) was purchased from Winkler (Santiago, Chile), while pectin (Pec) from citrus peel (Galacturonic acid ≤ 65%) was donated by Quimatic (Santiago, Chile). *Limosilactobacillus reuteri* DSM 17938 was isolated from a commercial product (Protectis baby drops, BioGaiga AB, Stockholm, Sweden). Man Rogosa Sharpe (MRS) agar, MRS broth, and DIFCO bacteriological peptone were supplied by Dilaco (Santiago, Chile). All chemical reagents were of analytical grade. All samples were prepared with ultrapure water from the UV Direct-Q 5 purification system (Millipore, Molsheim, France). All reagents and laboratory equipment were sterilized before use.

### 2.2. Preparation of Limosilactobacillus reuteri Inoculum

In the present study, *Limosilactobacillus reuteri* DSM 17,938 cells were isolated from a commercial product (Protectis baby drops, BioGaiga AB, Stockholm, Sweden) and cultured in MRS agar selective medium at 37 °C for 48 h under anaerobic conditions [6]. The bacteria isolated from the commercial sample were separated by centrifugation at 3800× *g* (4 °C, 15 min), washed twice with a phosphate buffer solution (PBS) (0.01 M, pH 7.4), inoculated in MRS broth, and incubated at 37 °C for 18 h to obtain a 9 log CFU/mL concentration of colony-forming units. Finally, bacteria were suspended in PBS for encapsulation in a double emulsion. Fresh cultures of Lr were used for each experiment.

### 2.3. Preparation of Multilayer Double Emulsion

#### 2.3.1. Preparation of Double Emulsion Containing *Limosilactobacillus reuteri*

The W_1_/O/W_2_ emulsions were prepared by a two-step emulsification process [31]. The W_1_ of the single emulsion consisted of an Lr suspension in PBS supplemented with 2% *w*/*v* chicory-derived fructooligosaccharide (FOS). The FOS is considered to be a prebiotic that can stimulate Lr growth [32]. The oil phase (O) consisted of 65 mL Belmont brand canola oil mixed with a 5 mL Span 80/Tween 80 mixture in a 4:1 ratio (HLB, 6.4), prepared by magnetic shaking at 400 rpm and 25 °C. The single emulsion (Lr/O) was 30 mL of Lr and 70 mL of O mixed with a rotor-stator homogenizer (T25 Ultra-turrax, IKA, Werke, Germany) at 10,000 rpm for 5 min. The ratios of the emulsifier (4:1) and aqueous to oil phase (30:70) were determined in preliminary studies based on emulsion stability (Supplementary Table S1). Afterward, 30 mL of Lr/O was combined with 70 mL of an outer aqueous phase containing 17 g/L Cas or 20 g/L WPI in PBS. The mixture was homogenized at 8000 rpm for 5 min to produce the double emulsion (Lr/O/Cas or Lr/O/WPI). The 30:70 ratio of primary emulsion to external aqueous phase was employed due to its higher physical stability (Supplementary Figure S1). The Cas and WPI concentrations used in the external aqueous phase of the double emulsion corresponded to the minimum saturation concentration, as determined through ζ-potential measurements (Supplementary Figure S2a,b).

#### 2.3.2. Formation of Multilayer Double Emulsions

**Figure 1:**
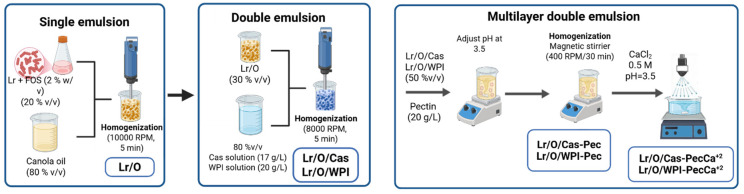
Schematic of multilayer double emulsion formation process.

Double emulsions were coated with a pectin layer according to the layer-by-layer electrostatic deposition technique [33], as illustrated in Figure 1. Each double emulsion was mixed in a 1:1 ratio with a Pec (2% *w*/*v*) aqueous dispersion in a magnetic stirrer at 400 rpm and 25 °C. It was previously determined that a 1% (*w*/*v*) Pec concentration was the minimum necessary to saturate the surface of the droplets of the double emulsions (Supplementary Figure S2c). The pH of the mixture was adjusted to 3.5 to promote electrostatic deposition of Pec on the outer layer of the Cas or WPI in the double emulsion droplets (Lr/O/Cas-Pec or Lr/O/WPI-Pec) (Supplementary Figure S3a,b). Stirring was maintained for 30 min.

In addition, the outer Pec layer of the double emulsions was subjected to an ionic gelation process to enhance the protection of Lr further. An Lr/O/Cas-Pec or Lr/O/WPI-Pec dispersion (20 mL) was sprayed at a distance of 98 cm on 100 mL of calcium chloride solution (0.5 M, pH 3.5) with an electrostatic air-assisted sprayer without an electrostatic charge (SC-ET, Electrostatic Spraying Systems Inc., Athens, GA, USA). The dispersion was maintained under magnetic shaking for 30 min to ensure the total gelation of the alginate layer [12].

The formed cross-linked microcapsules (Lr/O/Cas-PecCa^+2^ and Lr/O/WPI-PecCa^+2^) were filtered on Whatman Grade 1 paper (11 μm pore size) and rinsed five times with sterile peptone water (pH 3.5). They were then sealed in sterile tubes and stored at 4 °C. Table 1 lists the codes for the treatments under analysis.

### 2.4. Characterization of Emulsions

#### 2.4.1. Microstructure and Droplet Size

Emulsions were diluted (1:5) in PBS and analyzed with an optical microscope (Olympus BX53, Olympus Optical Ltd., Tokyo, Japan) at 40× magnification. Images were captured with a digital camera and analyzed with ImageJ software (version 1.53t). At least 10 regions of the slide from each emulsion type were randomly evaluated in triplicate to verify sample homogeneity and ensure adequate representation of droplet morphology and size distribution. Droplet diameter (D_(4,3)_) was calculated by Equation (1) [22]

(1) $$D_{\left(4,3\right)}=\frac{\sum n_{i}d_{i}^{4}}{\sum n_{i}d_{i}^{3}}$$

where *n_i_* is the number of particles in each size class per unit volume of emulsion and *d_i_* the diameter of the particles in each size class. The droplet size distribution (Span) was determined by Equation (2):

(2) $$Span=\frac{d\left(0.9\right)-d\left(0.1\right)}{d\left(0.5\right)}$$

where *d*(0.1), *d*(0.5) and *d*(0.9) are the volume-weighted sizes below which 10%, 50%, and 90% of the particles are counted, respectively. Analyses were performed in triplicate.

#### 2.4.2. Zeta Potential

The ζ-potential of the emulsified systems was measured by dynamic light scattering with a Zetasizer Nano ZS (Malvern Instruments Ltd., Worcestershire, UK). The emulsions were diluted 100 times with ultrapure water before analysis. Analyses were performed in triplicate.

#### 2.4.3. Viability of Microencapsulated *Limosilactobacillus reuteri* and Encapsulation Efficiency

To evaluate probiotic viability, 1 mL of the emulsion was centrifuged at 14,500× *g* for 10 min to disrupt the system, followed by serial dilution in peptone water and surface plating on MRS agar [12]. Incubation was performed at 37 °C for 48 h under anaerobic conditions. Viable counts were expressed as log CFU/mL.

Encapsulation efficiency was defined as the percentage of Lr trapped in the internal aqueous phase of the double emulsion. The encapsulation efficiency of the Lr/O/Cas and Lr/O/WPI emulsions was evaluated by Equation (3):

(3) $$EE\left(\%\right)=\left(\frac{Total\;live\;cells\;after\;emulsificiaction-unencapsulated\;live\;cells\;(\frac{\mathrm{CFU}}{\mathrm{mL}})}{Total\;live\;cells\;before\;emulsificaction\;(\frac{\mathrm{CFU}}{\mathrm{mL}})}\right)\times \;100$$

Total probiotics after emulsification refer to the count of viable microorganisms released from microcapsules by centrifugation. Non-encapsulated probiotics are the direct count of viable probiotics in the emulsion. Total probiotics before emulsification represent the initial probiotic count [34].

### 2.5. Stability of Multilayer Double Emulsions During Storage

To evaluate their stability, 10 mL of the single, double, and multilayer emulsions were placed in sterile tubes and stored at 4 °C. At different times (0, 7, 14, 21, and 28 d), 1.5 mL of each emulsion was transferred to sterile vials and centrifuged at 14,500× *g* at 4 °C for 10 min to break the emulsions and release the encapsulated bacteria. The total count of the viable cells in each emulsion system was determined as described in Section 2.4.3. All tests were performed in triplicate.

### 2.6. Thermal Tolerance of Microencapsulated Limosilactobacillus reuteri

To assess thermal stability, 10 mL of the sample (free or encapsulated bacteria) was placed in sterile vials and immersed in a water bath at 95 °C for 30 min [35]. Samples were cooled in an ice bath immediately after heating and analyzed for viable counts. Surviving bacteria were counted as described in Section 2.4.3. All tests were performed in triplicate.

### 2.7. Simulated In Vitro Digestion

The simulated gastric fluid (SGF) and simulated intestinal fluid (SIF) were prepared according to the standardized INFOGEST static digestion protocol [36]. Equal concentrations of free and encapsulated Lr cells in a single, double, and multilayer double emulsion and crosslinked microcapsules were diluted in a buffer solution (5 mM PBS, pH 7) [37]. Free and microencapsulated microorganisms (15 mL) were sequentially added to the SGF (45 mL, pH 3) for 2 h and then to the SIF (45 mL, pH 7.0) for an additional 2 h.

The SGF was prepared by mixing the stock solution with 3 mL pepsin (2000 U/mL), 15 µL CaCl2 (0.3 M), 0.3 mL HCl, and 0.45 mL distilled water, and the pH was adjusted to 3.0. The mixture was incubated at 37 °C with constant shaking at 50 rpm for 2 h. Afterward, SGF was mixed with 16.5 mL SIF containing 7.5 mL pancreatin solution (800 U/mL), 60 µL CaCl2 (0.3 M), 225 µL NaOH, 1.97 mL distilled water, and 3.75 mL bile extract. This mixture was incubated at 37 °C for an additional 2 h with constant shaking (50 rpm). During digestion, samples were collected every 30 min in the gastric and intestinal phases. The viability of free and microencapsulated cells was determined according to the procedure described in Section 2.4.3

### 2.8. Stability of Microencapsulated Limosilactobacillus reuteri Under Combined High Temperature and Gastrointestinal Conditions

To mimic the conditions that microencapsulated probiotic bacteria encounter when added to a baked product, Lr viability was assessed in two consecutive stages, that is, a thermal process and a simulated in vitro digestion step. The procedures outlined in Section 2.6 and Section 2.7 were followed for each stage. Finally, the viability of free and microencapsulated cells was determined according to the procedure described in Section 2.4.3.

### 2.9. Statistical Analysis

All results were subjected to analysis of variance (ANOVA) with Statgraphics Centurion version XVI software (Bitstream, Cambridge, UK). Significant differences between mean values (*p* ≤ 0.05) were determined by Tukey’s test. The results were reported as the mean value ± standard deviation. The analyses were performed in triplicate, and the results of the microencapsulation were calculated with the averages of three independent experiments.

## 3. Results and Discussion

### 3.1. Characterization of Multilayer Double Emulsions

#### 3.1.1. Microstructure, Droplet Size Distribution, and Zeta Potential

**Figure 2:**
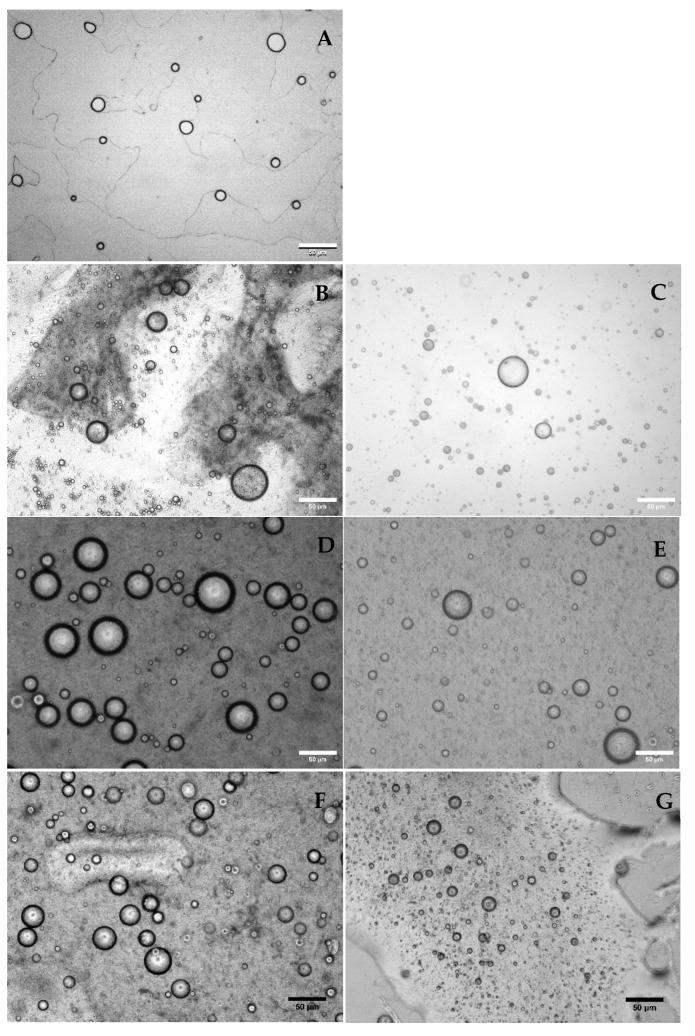
Morphology of different emulsions containing *Limosilactobacillus reuteri*: single emulsion (**A**); double emulsions: Lr/O/Cas (**B**) and Lr/O/WPI (**C**); double emulsions coated with a pectin layer: Lr/O/Cas-Pec (**D**) and Lr/O/WPI-Pec (**E**); double emulsions coated with a cross-linked pectin layer: Lr/O/Cas-PecCa^2+^ (**F**), and Lr/O/WPI-PecCa^2+^ (**G**). (scale bar = 50 µm).

Figure 2 shows the microstructure of the different emulsified systems that encapsulate Lr. Figure 2A displays the microstructure of Lr/O in which droplets of the aqueous phase containing Lr cells are dispersed in the oil phase of the emulsion. Figure 2B,C confirm the formation of a double emulsion using Cas and WPI in the external aqueous phase. These emulsions exhibit a “bubble in bubble” structure that is a characteristic of double emulsions [38]. In this structure, the oil phase droplets containing multiple aqueous phase droplets with probiotic bacteria are evenly distributed in the external aqueous phase. Several studies have confirmed these characteristics in probiotic bacteria encapsulated in double emulsions [30,38,39].

The thicker interfacial structure observed in the double emulsions, shown in Figure 2D,E, corresponds to the Pec layer formed through an electrostatic deposition process on the surface of Cas and WPI. Figure 2F,G show the double emulsions with this interfacial structure after being subjected to a cross-linking process with calcium ions.

The physicochemical properties of different emulsified systems are summarized in Table 2. Increased emulsion complexity significantly affected their properties (*p* ≤ 0.05). Droplet sizes of the double emulsions (Lr/O/Cas, 38.9 μm; Lr/O/WPI, 34.1 μm) were larger than for the single emulsions (Lr/O, 13.5 μm), particularly when using Cas in the external phase. The larger size in the Lr/O/Cas emulsion was probably caused by the higher molecular weights of Cas components (α-s1 casein: 22–23.7 kDa; β-casein: 24 kDa) [40] compared with WPI (β-lactoglobulin: 18.4 kDa; α-lactoalbumin: 14.178 kDa) [41]. Higher molecular weight proteins adsorb more slowly onto droplets and produce larger sizes. The larger droplet size in double emulsions was primarily attributable to adding a new aqueous phase (Cas or WPI) in single emulsions. Other studies have shown a similar trend, indicating that Lr encapsulated in a double emulsion was approximately three times the size compared with a single emulsion [7,12].

The droplet size of the double emulsion remained largely unchanged by the formation of the Pec layer (Lr/O/Cas-Pec and Lr/O/WPI-Pec) or the cross-linking with calcium ions (Lr/O/Cas-PecCa^+2^ and Lr/O/WPI-PecCa^+2^), regardless of the protein used in the aqueous phase. This finding is consistent with other studies on encapsulated *Lactobacillus casei* C24 in double emulsions coated with alginate and chitosan through layer-by-layer electrostatic deposition [31].

The formation of interfacial layers through the electrostatic deposition process was influenced by the degree of electrostatic interactions between Cas and Pec, as well as between WPI and Pec. The amount of Pec adsorbed at the interface was minimal, resulting in a very thin layer. The ionic cross-linking of the pectin layer with calcium ions led to cross-linked microcapsules that were slightly smaller than the double emulsion droplets, although this decrease was not significant (Figure 2F,G). This size reduction could arise from the formation of a more cohesive surface structure, which partially releases the internal aqueous phase of the microcapsules [42].

The coefficient of variation (CV) for droplet size, between replicates for each emulsified system, ranged from 21% for Lr/O to 7% for Lr/O/WPI-PecCa^+2^. The data dispersion relative to the mean was most significant for simple emulsions (21%). Still, it decreased for double emulsions (6–12%) and multilayer double emulsions (7–9%), indicating that the microencapsulation method used can be considered reproducible and reliable.

Table 2 shows that all emulsions were polydisperse with a wide droplet size distribution (Span: 0.5–0.8). The broad size distribution can be caused by shaking during emulsion formation (10,000 rpm for 5 min) with the rotor-stator homogenizer, which creates an unsteady flow. However, forming a Pec layer on double emulsion droplets reduced their polydispersity to 0.5. Shaking during this process likely promoted the coalescence of smaller droplets, resulting in a more uniform size distribution and a decrease in overall droplet size [25].

The ζ-potential is crucial for assessing colloidal stability in emulsions, as it indicates surface charge and resistance to aggregation. The single emulsion (Lr/O) showed a negative ζ-potential of −24.1 mV, which was linked to the presence of O. The formation of the double emulsion (Lr/O/Cas or Lr/O/WPI) had a positive ζ-potential shift and Cas and WPI altered the surface charge to +23.0 and +27.8 mV, respectively, which revealed their presence at the interface. The higher charge of the Lr/O/WPI emulsion can be attributed to increased WPI adsorption at the interface, which is due to the low molecular weight of its components and the higher charge density of WPI. Pectin adsorption onto double emulsion surfaces resulted in charge inversion, which led to negative ζ-potential values of −34.5 mV for Lr/O/Cas-Pec and −35.7 mV for Lr/O/WPI-Pec. This means that pectin interacted electrostatically with proteins at the interface to create a secondary stabilizing layer. Cross-linking the Pec layer with calcium ions significantly reduced (*p* < 0.05) the charge of the Lr/O/Cas-PecCa^2+^ and Lr/O/WPI-PecCa^2+^ microcapsules by partially neutralizing the Pec carboxylate groups (-COO^−^), which decreased electrostatic repulsion and created a more compact structure. Considering these results, it can be stated that the Lr/O/Cas-Pec and Lr/O/WPI-Pec emulsions presented the highest physical stability, due to their higher absolute ζ-potential value. The droplets of these emulsions exhibit a high repulsive electrostatic force, preventing the aggregation and migration of the emulsion droplets [43].

#### 3.1.2. Viability of Microencapsulated *Limosilactobacillus reuteri* and Encapsulation Efficiency

Table 3 displays the viability of Lr cells in various fresh emulsions. The encapsulation process reduced viability by 1.1 log CFU/mL in single emulsions and 0.6–0.7 log CFU/mL in double emulsions, regardless of the emulsifier used. The higher homogenization speed (10,000 rpm) for single emulsions can explain the greater loss of viability compared with double emulsions (8000 rpm). The formation of a pectin layer on the double emulsion using layer-by-layer electrostatic deposition (Lr/O/Cas-Pec and Lr/O/WPI-Pec) and subsequent ionic gelation (Lr/O/Cas-PecCa^+2^ and Lr/O/WPI-PecCa^+2^) did not significantly affect Lr viability (*p* ≤ 0.05). Despite the mechanical agitation involved, this slight decrease revealed a gentle encapsulation process. The decrease in viability was lower than findings reported by other authors for *Lactobacillus salivarius* in emulsions with protein layers and cross-linked pectin [23]. Encapsulation efficiency of Lr in double emulsions (Lr/O/Cas and Lr/O/WPI) achieved 94.2% and 92.7%, respectively. Similar high efficiency values have been noted for *Lactobacillus plantarum* (97.8%), *Lactobacillus rhamnosus* (95.9%), and *Akkermansia muciniphila* (97.5%). This high efficiency is attributed to the structure of double emulsions, which contain probiotic cells in water droplets within an oil phase, thereby preventing cell migration to the outer aqueous phase [22,30].

### 3.2. Storage Stability of Microencapsulated Limosilactobacillus reuteri

**Figure 3:**
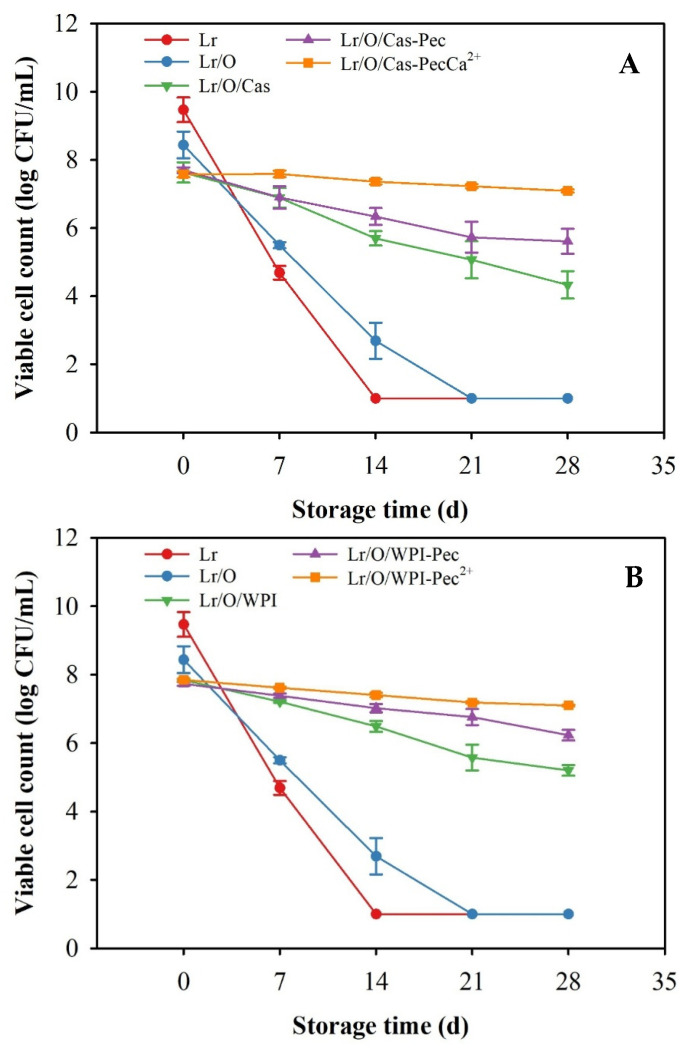
Viability of free (control) and microencapsulated *L. reuteri* in various emulsified systems during 28 d storage at 4° C when using sodium caseinate (Cas) (**A**) or whey protein isolate (WPI) (**B**) as a stabilizer. The mean value ± standard deviation of at least three independent measurements is included.

A high viable cell count of probiotic bacteria during food processing and storage is essential to achieve beneficial health effects [44]. The stability of microencapsulated Lr in emulsified systems with Cas or WPI was evaluated at 4 °C during 28 d (Figure 3). Unencapsulated and encapsulated Lr in single emulsion (Lr/O) exhibited a rapid decrease in viable cell counts, and complete inactivation (<1 log CFU/mL) occurred at 14 and 21 d, respectively. The enhanced viability of the probiotic in the Lr/O emulsion can be attributed to the presence of the FOS prebiotic in the aqueous phase, which supports bacterial growth [32]. Additionally, the oil phase may act as a protective barrier for the probiotic.

*Limosilactobacillus reuteri* improved viability in complex emulsified systems compared with single emulsions. In Lr/O/Cas and Lr/O/WPI double emulsions, viability gradually decreased, with bacterial counts after 28 d of 4.3 ± 0.39 log CFU/mL and 5.2 ± 0.15 log CFU/mL, respectively. The external aqueous phase (Cas and WPI) significantly protected Lr during storage. However, emulsions with WPI (Figure 3B) maintained greater viability during storage than those with Cas as an emulsifier. The higher protection shown by WPI could be attributed to the denser and more compact interfacial structure formed in the double emulsion (Figure 2B,C). The thicker interfacial layer in double emulsions provides greater stability against droplet aggregation and coalescence, as well as elasticity against deformation [45]. Also, the greater stability of probiotic cells observed in emulsions with WPI may be related to the higher ζ-potential (27.8 mV) compared to emulsions with Cas (23.0 mV). During storage, emulsions with WPI may have experienced less aggregation and interfacial destabilization due to the greater repulsive electrostatic force between droplets. The WPI is a low-molecular-weight protein with a globular structure that enhances adsorption and interface coverage, reduces water diffusion, and delays droplet coalescence. Proteins undergo a conformational change after adsorption on the surface of oil droplets to maximize the number of interactions, and the extent of these conformational changes is greater when the oil is more nonpolar [45,46]. In contrast, Cas has a flexible and open structure that promotes micelle formation and a less dense interface. This encourages greater diffusion of the internal phase (W_1_) to the exterior during storage, thus increasing cell exposure to the environment.

The formation of a pectin surface layer on double emulsions further enhanced the protection of probiotics during storage. After the storage period, probiotic counts were 5.61 ± 0.37 log CFU/mL for Lr/Lr/Lr/O/Cas-Pec and 6.23 ± 0.15 log CFU/mL for Lr/Lr/Lr/O/WPI-Pec. The electrostatic interactions between proteins (Cas or WPI) and Pec created a thicker interfacial structure, reducing the mobility of the internal aqueous phase and thereby improving bacterial protection.

However, the maximum viability of the probiotic was achieved when encapsulated in a Pec-coated double emulsion, which was subjected to ionic crosslinking with calcium (Lr/O/WPI-PecCa^2+^ and Lr/O/Cas-PecCa^2+^). In these cross-linked microcapsules, viable cell counts >7.0 log CFU/mL were maintained at the end of storage, regardless of the type of protein used as an emulsifier in the initial emulsions. The cross-linked Pec layer formed through the ionic crosslinking process was denser, stiffer, and had lower permeability, which more effectively reduced the migration of the aqueous phase and the loss of probiotic viability. Previous studies have demonstrated that encapsulation enhances the viability of probiotics during storage. For example, *Lactobacillus acidophilus* JYLA-191, encapsulated in a double network emulsion gel with whey protein concentrate, xanthan gum, and k-carrageenan, remained viable for 6 wk at 4 °C [46]. This gel helped regulate cell membrane fluidity and stress sensitivity. In addition, *L. casei* ATCC 393 and *L. rhamnosus* also maintained viable counts >7 log CFU/mL in Pec microgels after 42 d at 4 °C [47].

### 3.3. Thermal Tolerance of Free and Microencapsulated Limosilactobacillus reuteri

**Figure 4:**
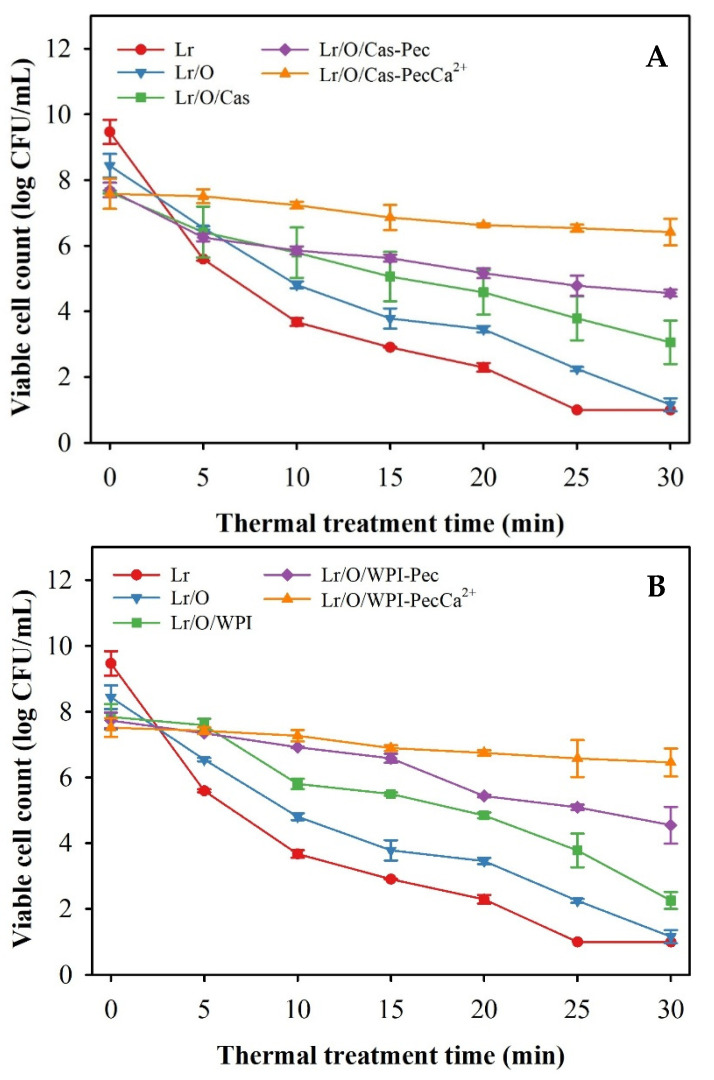
Viability of free (control) and microencapsulated *L. reuteri* (log CFU/mL) during heat treatment (95 °C, 30 min), using Cas (**A**) or WPI (**B**) as a stabilizer. The mean value ± standard deviation of at least three independent measurements is included.

It is essential to understand the behavior of microencapsulated probiotics during the baking process to produce baked goods with probiotics. The thermal tolerance of microencapsulated Lr was tested at 95 °C for 30 min, which simulated the conditions in the center of a loaf during baking (Figure 4). Heat treatment had a significant impact on the viability of free and microencapsulated Lr in various emulsified systems. Non-encapsulated cells were the most severely affected, rapidly losing viability and becoming completely inactivated within 25 min. All emulsified systems exhibited a protective capacity against temperature, which improved with increasing interfacial complexity. After 30 min of heat treatment, Lr in the Lr/O emulsion showed no activity, but its viability increased significantly in double emulsion: Lr/O/Cas (3.1 log CFU/mL), Lr/O/WPI (2.3 log CFU/mL) and when the double emulsions were coated with pectin: Lr/O/Cas-Pec (4.6 log CFU/mL), Lr/O/WPI-Pec (3.2 log CFU/mL). The Lr/O/Cas double emulsion was more effective than Lr/O/WPI in protecting Lr from temperature, achieving viable count reductions of 4.6 log CFU/mL and 5.6 log CFU/mL, respectively, at the end of the heat treatment. However, the formation of a pectin layer on the double emulsions provided greater heat protection, achieving a viable count reduction of 3.1 log CFU/mL, regardless of the type of protein used as a stabilizer in the emulsions. A similar protective effect has been described for a double emulsion with *L. rhamnosus* after a spray drying process. The inclusion of probiotics in the internal aqueous phase reduced exposure to drying air and protected them from thermal stress. Additionally, prebiotics in the inner aqueous phase enhanced the survival of probiotics [38].

*Limosilactobacillus reuteri* in microcapsules with a cross-linked Pec layer (Lr/O/WPI-PecCa^+2^) reached the highest viability (6.4 log CFU/mL) an the lowest reducction in viable counts (1.1 log CFU/mL) at the end of the heat treatment; this was similar to emulsified systems using Cas as an emulsifier (Figure 4A).

As previously mentioned, the development of a complex interfacial structure in these emulsions involving an external protein-based aqueous phase, a Pec layer formed through electrostatic deposition, and ionic gelation created a more effective protective barrier against the heating medium, which significantly enhanced the thermal protection of microencapsulated Lr. For these reasons, the cross-linked microcapsules (Lr/O/WPI-PecCa^2+^ and Lr/O/Cas-PecCa^2+^) exhibited the highest thermal protection among these emulsified systems. A similar situation has been described for *L. casei* NCDC-298 microencapsulated in calcium alginate. These authors report that the probiotic exhibits high thermal stability when heated at 55, 60, or 65 °C for 20 min, due to a reduction in the rate of water diffusion into the interior of the alginate beads [48]. Similarly, other authors have found that a cross-linked pectin layer on an emulsion using Cas or WPI as an emulsifier significantly improved the viability of *L. salivarius* NRRL B-30514 after 15 s at 73 °C [23]. Other studies have investigated the effect of baking temperatures on the viability of different probiotic strains incorporated into bread. Zhang et al., 2018, reported a decrease in viable cell counts of *Ltp. plantarum* from 9 log CFU/g to 4 log CFU/g after baking at 175 °C for 6 min [49]. Ezekiel et al., 2020, confirmed that after baking white pan bread at different temperature (180 °C for 30 min; 220 °C for 20 min; 250 °C for 15 min), cell-free *Lactobacillus rhamnosus* did not survive; however, the viability of the probiotic encapsulated in sodium alginate beads coated with different layers of biopolymers ranged between 4.94 and 9.19 log CFU/g, confirming that the three-layer capsules better protected the probiotic from heat stress [50].

It is important to note that the cross-linked microcapsules maintained a viable cell count of Lr >6 log CFU/mL after undergoing heat treatment. This is the minimum recommended level that probiotic-supplemented foods should have at the time of consumption to confer health benefits [10].

### 3.4. In Vitro Digestion of Microencapsulated Limosilactobacillus reuteri

**Figure 5:**
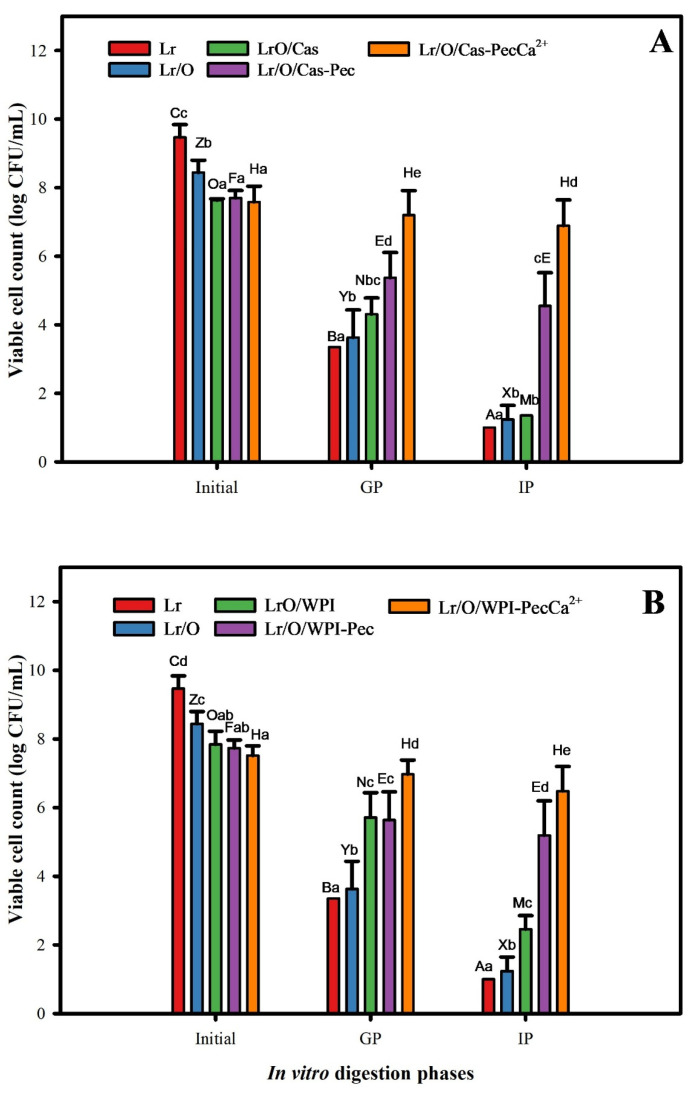
Viability of free (control) and encapsulated *L. reuteri* (log CFU/mL) when using Cas (**A**) or WPI (**B**) as a stabilizer during GP (gastric phase: 2 h, pH 3.0), and IP (intestinal phase: 2 h, pH 7.0) according to the standardized INFOGEST static digestion protocol. The mean value ± standard deviation of at least three independent measurements is included. Different uppercase letters in the same treatment (encapsulated or free cells) indicate statistical significance (*p* ≤ 0.05). Different lowercase letters in the same phase of digestion indicate statistical significance (*p* ≤ 0.05).

It is crucial to determine the viability of probiotics during gastrointestinal transit because of their beneficial effects on intestinal health [51]. Figure 5 illustrates the protective effect of emulsion structures on encapsulated Lr cells during simulated gastric and intestinal phases. There was a significant decrease (*p* ≤ 0.05) in the viability of both free and encapsulated cells after the gastric phase (GP) (2 h, pH 3.0). The most significant reductions in viability were noted in free cells (Lr, 6.1 log CFU/mL) and encapsulated cells in single emulsions (Lr/O, 4.8 log CFU/mL). These results demonstrate the sensitivity of Lr to the acidic conditions of the gastric phase (pH 3.0 and pepsin) [52]. Although *Lactobacilli* are generally considered tolerant to acidic conditions, a significant reduction in viability during the gastric phase has also been observed. It has been suggested that direct exposure to low pH and high oxygen levels during gastric digestion is responsible for the decreased viability [53].

For double emulsions, viability decreased by 3.3 log CFU/mL for Lr/O/Cas and 2.1 log CFU/mL for Lr/O/WPI. The smaller reduction in viability observed in Lr/O/WPI emulsions could be due to its higher surface charge (27.8 mV vs. 23.0 mV) (Table 2). The formation of a denser surface layer, caused by higher adsorption of WPI, can have limited the diffusion of gastric fluid into the emulsion. In turn, the enhanced protection of double emulsions for probiotics can be attributed to the fact that cells are trapped in the internal aqueous phase, which isolates them from gastric acids and enzymes [39]. Additionally, the protein layer of the emulsion offers protection against gastric fluids due to its buffering capacity [54]. On the other hand, the electrostatic deposition of pectin on the protein surface layer in the emulsions (Lr/O/Cas-Pec and Lr/O/WPI-Pec) contributed to further maintaining the viability of Lr, reaching a decrease in viability of 2.1–2.3 log CFU/mL, regardless of the emulsifier used. This finding is consistent with those reported by other authors, who indicated that coating indigestible pectin reduces the hydrolysis of the protein layer and demulsification [23,55]. The best protection against acidity was provided by Lr/O/Cas-PecCa^+2^ and Lr/O/WPI-PecCa^+2^, which decreased viability by only 0.4 and 0.5 log CFU/mL, respectively. The ionic cross-linking of calcium ions with pectin creates a denser calcium pectate layer, which reduces acid diffusion into the microcapsules [56]. A similar effect was noted for *L. salivarius* in a pectin-coated secondary emulsion under gastrointestinal conditions [23].

During the intestinal phase (IP), viable cell counts significantly decreased (*p* ≤ 0.05), with Lr, Lr/O, Lr/O/Cas, and Lr/O/WPI reaching reductions of 8.5, 7.2, 6.3 and 5.4 log CFU/mL, respectively, by the end of the phase. The reduction in viable cell counts in the free cells and the single emulsion is likely due to the direct action of bile salts, as they can alter bacterial cell membranes, affecting their viability. In double emulsions, proteases initially break down the protein layer. Then bile salts act as an emulsifier, destabilizing the oil phase into small droplets and promoting lipolysis by pancreatic lipases. Consequently, the probiotic cell membrane phospholipids are exposed to bile salts [23].

A more moderate reduction in counts of 3.1 and 2.5 log CFU/mL was observed in the Lr/O/Cas-CaPec and Lr/O/WPI-CaPec emulsions, respectively. This is attributable to the protection generated by the pectin layer formed on the surface of the double emulsion droplet, which delays the hydrolysis of the protein layer. However, under intestinal conditions (pH 7.0, protease, bile salts), Cas, WPI, and Pec become anionic (see Supplementary Figure S3), and the pectin layer can detach from the emulsion surface, which leads to hydrolysis and disintegration of the protein layer and exposes probiotic cells to bile salts [57]. In contrast, probiotic cells in the Lr/O/Cas-PecCa^+2^ and Lr/O/WPI-PecCa^+2^ microcapsules had the lowest viability reduction (0.7 and 1.0 log CFU/mL, respectively) and the highest viable counts (6.9 and 6.5 log CFU/mL, respectively) at the end of the intestinal phase because of the protective cross-linked pectin layer. The protection achieved by this encapsulation system was more effective than that achieved in a previous study that encapsulated *L. casei* in a double emulsion microcapsule coated with alginate-chitosan layers (Lc/O/Cas-A-Ch), where a reduction of 1.4 log CFU/g was achieved at the end of the simulated intestinal phase [31]. A similar effect was also noted for *L. salivarius* encapsulated in a secondary emulsion with a pectin layer cross-linked with calcium under gastrointestinal conditions [23]. Encapsulating *L. casei* in a double layer of cross-linked alginate has reduced the diffusion of bile salts into the microcapsules, thereby delaying contact with the probiotic bacteria [56].

Based on these results, it can be emphasized that the proposed microencapsulation system, which combines double emulsion, multilayer formation, and ionic cross-linking, creates an interfacial structure that effectively regulates the entry of gastric fluids into the microcapsules. This structure provides robust protection for probiotic cells, ensuring that viability levels remain above the threshold required for a product to be considered probiotic (>6 log CFU/g).

### 3.5. Stability of Microencapsulated Limosilactobacillus reuteri During Combined High Temperature and Gastrointestinal Conditions

**Figure 6:**
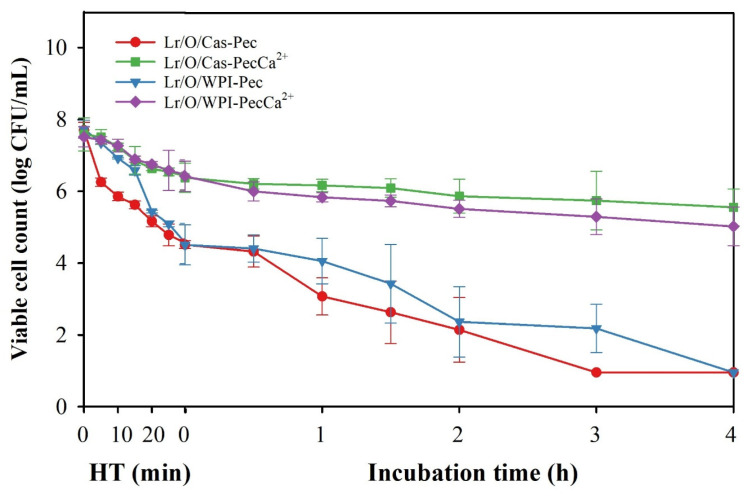
Combined effect of heat treatment (HT) and in vitro digestion on the viability of *L. reuteri* microencapsulated with an ionically cross-linked pectin layer. The mean ± standard deviation of at least three independent measurements is included.

Viable cell counts in the emulsified systems during thermal processing and simulated in vitro digestion are shown in Figure 6. Cell counts in all samples significantly decreased during both heat treatment (HT) and simulated digestion. Cells in Lr/O/Cas-Pec and Lr/O/WPI-Pec rapidly lost viability during heat treatment and became completely inactive by the end of the simulated digestion. This indicates that the interfacial structure of the pectin-coated double emulsions, regardless of the protein used, failed to protect the probiotic cells from the cumulative effects of temperature and gastrointestinal conditions. The thermal processing likely caused more significant structural changes in the proteins of these microcapsules, which promoted their agglomeration and altered their solubility. These structural changes, in turn, may have affected electrostatic interactions with the pectin layer, reducing its protective capacity against gastrointestinal fluids.

In contrast, the Lr/O/Cas-PecCa^2+^ and Lr/O/WPI-PecCa^2+^ microcapsules provided the greatest protection to Lr cells under stress conditions by maintaining high viable cell counts by the end of these stages (5.6 and 5.1 log CFU/mL, respectively). No significant differences were noted between Cas and WPI as emulsifiers. Cross-linking calcium ions with pectin resulted in a more compact interfacial structure in the emulsions, thereby significantly improving their protective capacity. This enhanced structure limited the movement of the heating medium into the microcapsules and reduced the diffusion of gastric fluids and bile salts during simulated digestion.

These results confirm that the proposed microencapsulation method has great potential for producing probiotic transport systems for the development of bakeable products and pasteurized vegetable juices. These microcapsules can protect probiotics from the high temperatures frequently used in food processing and during gastrointestinal transit, maintaining a high level of viability that allows them to exert their beneficial effects on consumer health. For their technological application, these microcapsules must be previously stabilized through processes such as freeze-drying or spray-drying to facilitate their incorporation into different food matrices.

## 4. Conclusions

The present study has demonstrated the potential of multilayer double emulsions (W_1_/O/W_2_) stabilized with protein-pectin complexes to encapsulate and protect *Limosilactobacillus reuteri* under technological and gastrointestinal stress. Both Cas and WPI effectively stabilized the double emulsion systems, generating a high net surface charge and ensuring high encapsulation efficiency (>92%). The use of WPI in the double emulsion protected the probiotic during refrigerated storage and under gastrointestinal conditions, while the use of Cas provided greater protection against high temperatures. However, the development of a more complex interfacial structure in the double emulsions significantly improved their ability to protect the probiotic against thermal stress and gastrointestinal conditions. Coating the emulsions with a pectin layer improved viability; however, ionic cross-linking of the pectin layer was more effective in increasing its protective capacity due to the formation of a denser layer with lower permeability. No significant differences were observed between Cas and WPI used as stabilizers in these types of multilayer double emulsions exposed to stress conditions. The protective capacity of these multilayered emulsified systems is remarkable, as viable probiotic counts of over 5 log CFU/mL can be achieved after undergoing consecutive stages of heat treatment and in vitro digestion.

The results demonstrate that the proposed microencapsulation method, which combines double emulsion, multilayer formation, and cross-linking, successfully creates an interfacial structure that effectively protects probiotic cells during exposure to sequential heat treatments and in vitro digestion. In these more realistic conditions, particularly in double emulsions with an ionically cross-linked pectin layer, the formed interfacial structure increased the thermal tolerance of the probiotic cells and effectively regulated the influx of gastric fluids into the microcapsules. This structure provides robust protection for probiotic cells, ensuring that viability levels remain above the threshold required for a product to be considered probiotic (>5 log CFU/g). These findings support the use of multilayer double emulsions as a promising platform to deliver probiotics in thermally processed or refrigerated food products with functional properties. Future studies should investigate the sensory and functional integration of these systems in real food matrices and validate their probiotic efficacy in vivo. It is necessary to confirm whether the protective capacity of the microcapsules is maintained after they are stabilized by freeze-drying or spray drying, to facilitate their incorporation into various food matrices.

## Figures and Tables

**Table 1:** Codes used for each type of emulsion.

Emulsion	Code
Simple emulsion (*Limosilactobacillus reuteri*/canola oil)	Lr/O
Double emulsion (*L. reuteri*/canola oil/sodium caseinate)	Lr/O/Cas
Double emulsion coated with pectin (*L. reuteri*/canola oil/sodium caseinate—pectin)	Lr/O/Cas-Pec
Double emulsion coated with cross-linked pectin (*L. reuteri*/canola oil/sodium caseinate—cross-linked pectin)	Lr/O/Cas-PecCa^+2^
Double emulsion (*L. reuteri*/canola oil/whey protein isolate)	Lr/O/WPI
Double emulsion coated with pectin (*L. reuteri*/canola oil/whey protein isolate—pectin)	Lr/O/WPI-Pec
Double emulsion coated with cross-linked pectin (*L. reuteri*/canola oil/whey protein isolate—cross-linked pectin)	Lr/O/WPI-PecCa^+2^

**Table 2:** Effect of pectin coating and cross-linking process on the physicochemical properties of multilayer double emulsions.

Code	d_43_ (µm)	Span	ζ-Potential (mV)
Lr/O	13.5 ± 2.9 ^a^	0.88 ± 0.03 ^d^	−24.1 ± 2.0 ^c^
Lr/O/Cas	38.9 ± 4.8 ^c^	0.69 ± 0.03 ^c^	23.0 ± 4.6 ^d^
Lr/O/Cas-Pec	39.6 ± 4.7 ^c^	0.62 ± 0.02 ^b^	−34.5 ± 2.2 ^a^
Lr/O/Cas-PecCa^+2^	38.0 ± 3.5 ^c^	0.55 ± 0.04 ^a^	−31.3 ± 0.9 ^b^
Lr/O/WPI	34.1 ± 2.2 ^b^	0.61 ± 0.03 ^b^	27.8 ± 1.6 ^d^
Lr/O/WPI-Pec	35.1 ± 2.2 ^b^	0.58 ± 0.02 ^ab^	−35.7 ± 1.5 ^a^
Lr/O/WPI-PecCa^+2^	34.1 ± 2.5 ^b^	0.55 ± 0.02 ^a^	−31.7 ± 0.7 ^b^

All values are mean ± standard deviation of three replicates. d_4,3_ De Brouckere mean diameters were calculated based on volume. Different superscript lowercase letters in the same column indicate significant differences between treatments (*p* ≤ 0.05).

**Table 3:** Effect of the microencapsulation process on viability.

Types of Emulsions	Viable Cell Count of *L. reuteri* (Log UFC/mL)
Lr	9.5 ± 0.5 ^c^
Lr/O	8.4 ± 0.6 ^b^
Lr/O/Cas	7.7 ± 0.4 ^a^
Lr/O/Cas-Pec	7.7 ± 0.1 ^a^
Lr/O/Cas-PecCa^+2^	7.6 ± 0.1 ^a^
Lr/O	8.4 ± 0.6 ^b^
Lr/O/WPI	7.8 ± 0.1 ^a^
Lr/O/WPI-Pec	7.7 ± 0.2 ^a^
Lr/O/WPI-PecCa^+2^	7.5 ± 0.7 ^a^

All values are mean ± standard deviation of three replicates. Different superscript lowercase letters in the same column indicate significant differences between treatments (*p* ≤ 0.05).

## Data Availability

The original contributions presented in the study are included in the article and Supplementary Material. Further inquiries can be directed to the corresponding author.

## Supplementary Materials

The following supporting information can be downloaded at: https://www.mdpi.com/article/10.3390/foods14142455/s1, Table S1: Effect of Span 80: Tween 80 ratio on Lr/O emulsion stability; Figure S1: Effect of Lr/O:W_2_ ratio on the stability of Lr/O/W_2_ double emulsion. (a) Size distributions, (b) size (d_43_), (c) Creaming index; Figure S2: Determination of the emulsifier saturation concentration (W2) in the double emulsion (W_1_/O/W_2_): (a) sodium caseinate, (b) whey protein isolate, and (c) saturation concentration of the pectin coating on the double emulsion.; Figure S3: Determination of the pH of maximum surface charge difference between: (a) Cas-Pec and (b) WPI-Pec.

## Abbreviations

The following abbreviations are used in this manuscript:

Lr	*Limosilactobacillus reuteri*
WPI	Whey protein isolate
Cas	Sodium caseinate
FOS	Fructooligosaccharide
GP	Gastric phase
IP	Intestinal phase
