# Supplementary material for: Multilayer Double Emulsion Encapsulation of Limosilactobacillus reuteri Using Pectin-Protein Systems
Source: Foods. 2025 Jul 12;14(14):2455. doi: 10.3390/foods14142455 (PMC12294583; doi:10.3390/foods14142455)
Supplement: Supplementary file 1 [file foods-14-02455-s001.zip › foods-3731868-supplementary.pdf]

Supplemental Information

Multilayer double emulsion encapsulation of *Limosilactobacillus reuteri* using pectin-protein systems

Kattya Rodriguez[1], Diego Catalán[1], Tatiana Beldarraín-Izanaga[2], Juan Esteban Reyes-Parra[1], Keyla Tortoló Cabañas[1], Marbelis Valdés Veliz[1] and Ricardo Villalobos-Carvajal[1,*]

Correspondence: r.villalobos@ubiobio.cl; Tel.: +56 422463095

Table S1. Effect of Span 80: Tween 80 ratio on Lr/O emulsion stability.

Span 80:Tween 80	HLB	Creaming index (%)	Droplet size (μm)
**3.75: 1.25**	7.0	33.67 ± 5.98 [bc]	32.11 ± 2.16 [c]
**4: 1**	6.4	10.60 ± 4.28 [a]	27.42 ± 0.91 [a]
**4.25: 0.75**	5.9	28.27 ± 4.69 [b]	28.94 ± 2.57 [b]
**4.5: 0.5**	5.4	38.20 ± 2.27 [cd]	29.92 ± 1.37 [b]
**4.75: 0.25**	4.8	41.53 ± 5.48 [d]	33.83 ± 1.87 [d]

All values are mean ± standard deviation of three replicates.
Different lowercase letters in the same column indicate significant differences between treatments ($p \leq 0.05$).

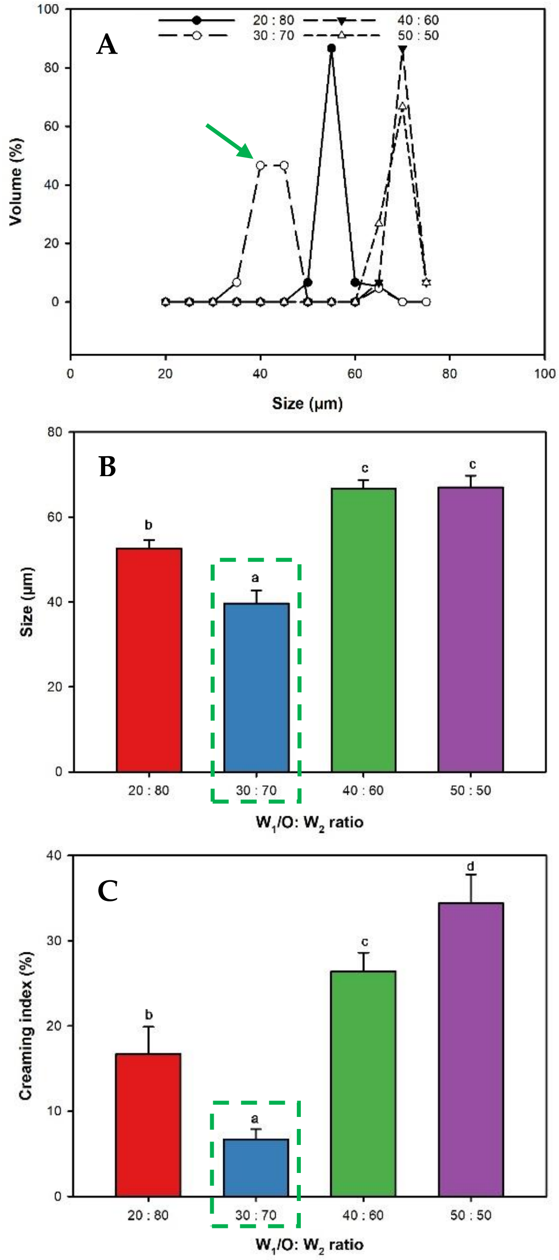

Figure S1. Effect of Lr/O:W_2 ratio on the stability of Lr/O/W_2 double emulsion. a) Size distributions, b) size (d_{43}), and c) Creaming index.

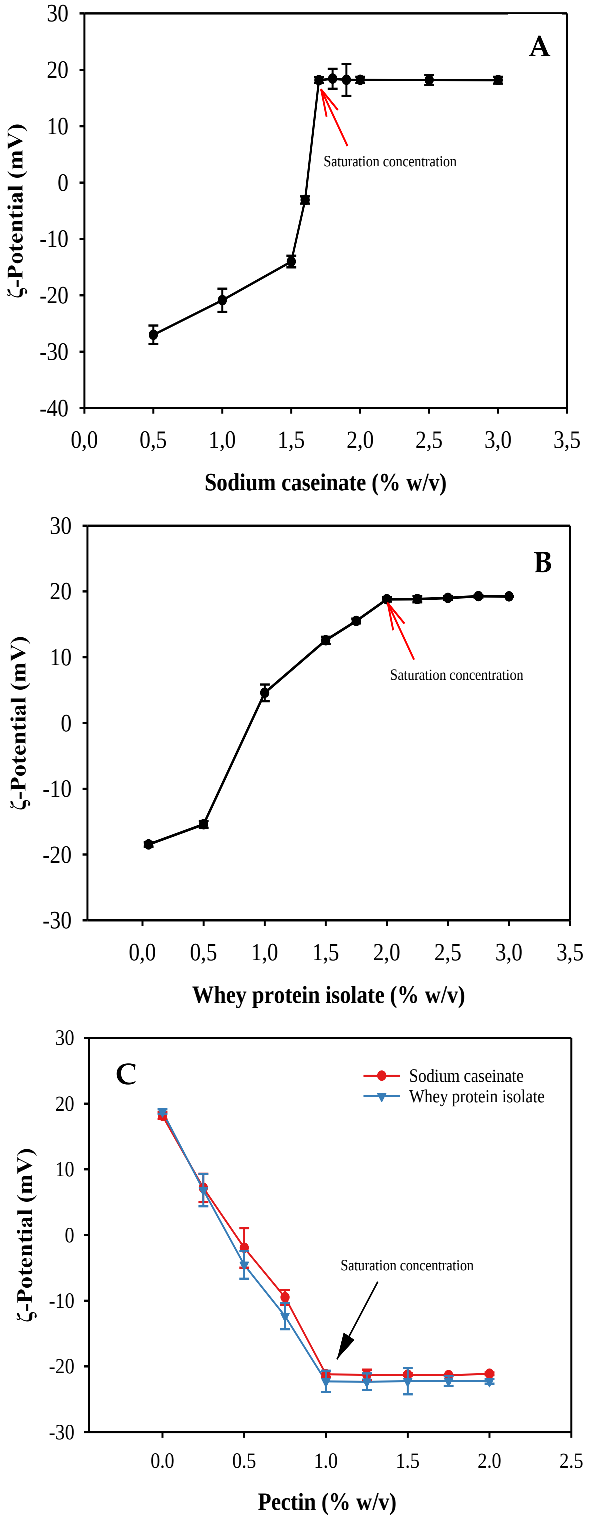

Figure S2. Determination of the emulsifier saturation concentration (W_2) in the double emulsion ($W_1/O/W_2$): a) sodium caseinate, b) whey protein isolate, and c) saturation concentration of the pectin coating on the double emulsion.

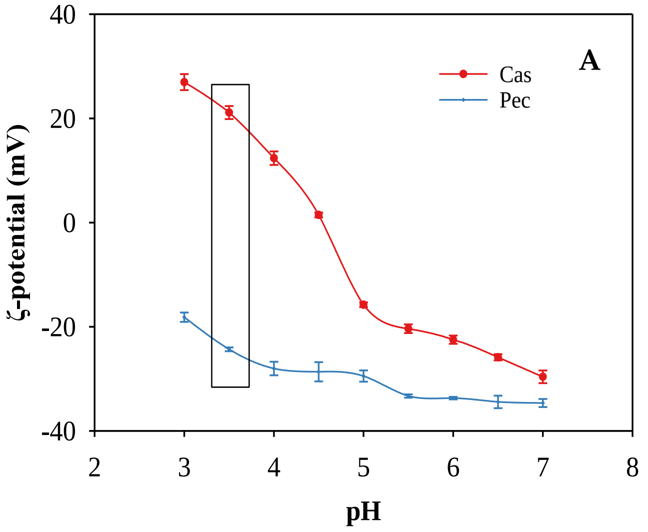

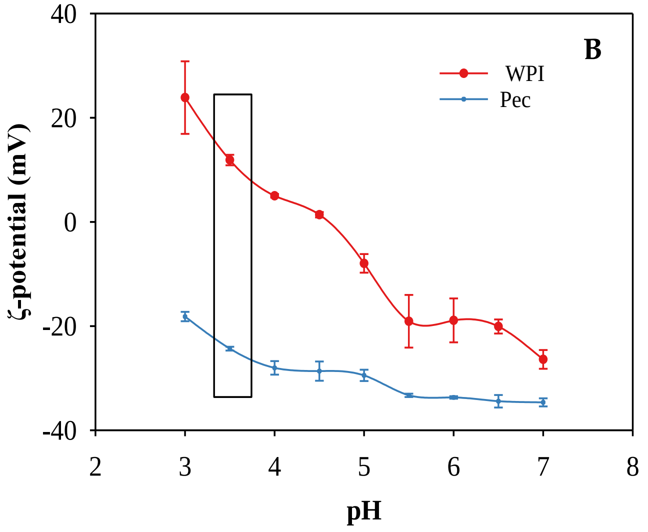

Figure S3. Determination of the pH of maximum surface charge difference between: a) Cas-Pec and b) WPI-Pec.
